# Airborne Optical Sectioning for Nesting Observation

**DOI:** 10.1038/s41598-020-63317-9

**Published:** 2020-04-29

**Authors:** David C. Schedl, Indrajit Kurmi, Oliver Bimber

**Affiliations:** Johannes Kepler University, Faculty of Engineering and Natural Sciences, Linz, 4040 Austria

**Keywords:** Population dynamics, Conservation biology

## Abstract

We describe how a new and low-cost aerial scanning technique, airborne optical sectioning (AOS), can support ornithologists in nesting observation. After capturing thermal and color images during a seven minutes drone flight over a 40 × 12 m patch of the nesting site of Austria’s largest heron population, a total of 65 herons and 27 nests could be identified, classified, and localized in a sparse 3D reconstruction of the forest. AOS is a synthetic aperture imaging technique that removes occlusion caused by leaves and branches. It registers recorded images to a common 3D coordinate system to support the reconstruction and analysis of the entire forest volume, which is impossible with conventional 2D or 3D imaging techniques. The recorded data is published with open access.

## Introduction

Civil drone applications increase radically world wide to support many areas, such search and rescue, remote sensing, delivery of goods, and wildlife observation^1^. With increasing numbers of civil drones, also new challenges arise that need to be addressed (e.g., ethics, sustainability, security, privacy)^2,3^.

Traditional bird census techniques^4^ that involve human volunteers might lead to a considerable amount of damage and disturbance to the animals’ habitat. They are also limited by safety, logistical, and financial constraints. Today, several technological options exist for monitoring bird ecology. Radar^5^, for instance, is widely used to study migratory patterns of birds and is particularly useful for observing birds which fly at high altitudes and in darkness. Automated acoustic sensor units^6^, as another example, are applied for species identification and perform equally well as human observers. Imaging sensors are often utilized for estimating population count^7^. The counting and observation of nesting birds, however, are an enormous challenge to ornithologists. Nests are often hidden under dense tree crowns and are neither visible from the the ground nor the air. Modern observation methods utilize aerial color and thermal imaging using camera drones^8–11^. But animals and nests occluded by branches, leaves, and shrubbery cannot be detected with conventional imaging techniques^5,6^.

**Figure 1:**
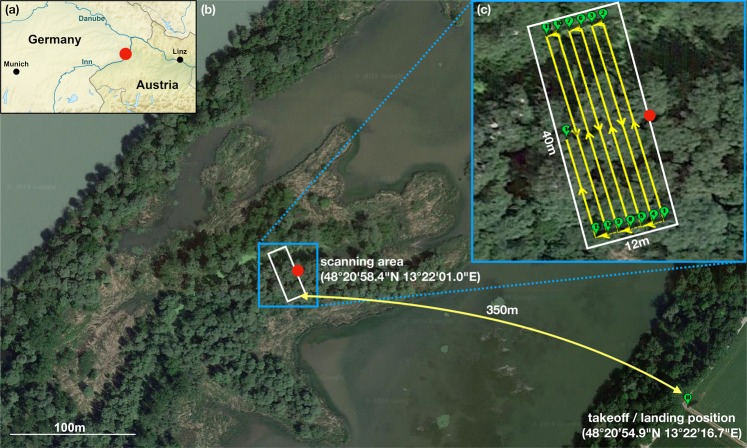
Field experiment at the wild life resort Lower Inn, Reichersberg located in Upper Austria (**a**). The largest heron colony in Austria nests within an isolated water meadow of the Inn river from March to July (**b**). A camera drone was used to autonomously scan an area of 40 × 12 m **(c**) at an altitude of 35 m above ground level/10 m to 15 m above the tree crowns. It captured a sequence of 130 high resolution RGB-thermal image pairs while flying a 7 min scanning pattern (**c**) before returning to the 350 m remote takeoff site (**b**). The red dot indicates a common position for better orientation. See also Supplementary Video. Copyright: (**a**) is made with Natural Earth; (**b**) and (**c**) contains imagery from Google Maps, GeoBasis-DE/BKG, GeoContent, Geoimage Austria, Maxar Technologies.

In this article, we present the results of a field experiment at the wild life resort Lower Inn, Reichersberg (Fig. 1b) that utilized a new synthetic aperture imaging technique, called airborne optical sectioning (AOS)^12–14^, to count herons at Austria’s largest colony during nesting season. Local ornithologists have to keep a running record of the population and the number of occupied nests for annual updates of the Upper Austrian Red List of Threatened Species^15^. All three observed species (i.e., black-crowned night herons *(Nycticorax nycticorax)*, little egrets *(Egretta garzetta)*, and grey herons *(Ardea cinerea)*) are categorized as endangered (EN). The remote nesting site is surrounded by wetlands and not easily accessible. Therefore, conventional camera drones have been applied for monitoring in the past. Thus far, however, aerial imaging was only possible during early spring and autumn (before and after the actual breeding season) when trees did not carry occluding leaves. Since the animals are not steady, the population could only be estimated by counting nests. During breading season (March - July), young and adult birds are more stationary in their nests and could directly be counted, but leaf occlusion prevents proper monitoring from the air and from the ground. We show that AOS is an adequate aerial imaging technology for capturing occluded birds and nests (during breeding season) that remain invisible to normal cameras or binoculars.

Synthetic aperture techniques approximate a theoretical wide-aperture sensor by computationally combining the signals of multiple small-aperture sensors or a single moving small-aperture sensor. Thus, wide-aperture signal characteristics can be achieved (i.e., increased resolution, depth-of-field, frame rate, contrast, and signal-to-noise ratio). This principle is applied in areas, such as radar^16–18^, radio telescopes^19,20^, microscopy^21^, sonar^22,23^, ultrasound^24,25^, LiDAR^26,27^, and optical imaging^28–35^.

Our drone was equipped with synchronized color (RGB) and thermal cameras and autonomously recorded a sequence of images at an altitude of 10 m to 15 m above the forest (Fig. 1). These images were then computationally combined to form the signal of a wide synthetic aperture which allowed slicing the forest optically from the ground to the tree crowns – similar to optical sectioning commonly used in high-NA microscopy. Browsing the resulting focal stacks allowed uncovering many hidden animals and nests. They could easily be located in the thermal signal, classified in the RGB signal, and visualized in a sparse 3D reconstruction of the forest.

Since full 3D reconstructions are not feasible for complex environments with significant occlusion, such as dense forests, AOS does not rely on feature matching in images (as required for photogrammetry^36,37^), or direct depth measurements (as for LiDAR^38–41^). Instead, it is a low-cost and efficient image-based technique that still supports 3D visualization. For occlusion removal, it relies on the statistical likelihood that enough bits of heat radiation or reflected light can be collected through a dense occluder volume when scanned over a wide directional range to form clear and occlusion free images at chosen focal distances^42^. Compared to reviewing sequential video sequences or single photographs, AOS makes nesting observation and bird counting much easier since it registers all recorded images to a common 3D coordinate system to support the image-based analysis of the entire forest volume.

## Results

The scanning path that outlines a synthetic aperture of 40 × 12 m (Fig. 1c) was defined prior to the flight. During flight, one RGB-thermal image pair was captured at 2 m steps along the path. This resulted in a total of 130 image pairs captured in around 7 min (ground speed of the drone was 0.7 ms^−1^).

**Figure 2:**
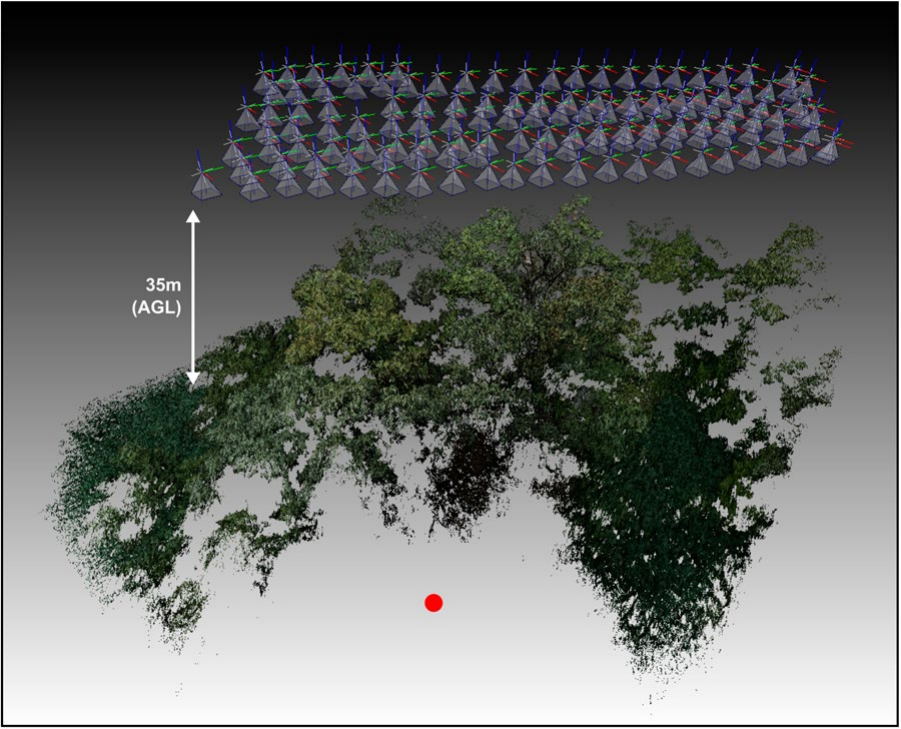
Images are recorded 35 m above ground level/10 m to 15 m above the tree crowns. The high-resolution RGB images were used for exact 3D pose estimation of the drone, and a sparse 3D reconstruction of the forest. The red dot indicates a common position for better orientation.

The high-resolution RGB images were used for exact 3D pose estimation of the drone (Fig. 2). Note, that GPS and internal inertia and compass sensors are too imprecise for this. The average visual pose-estimation error was 0.548 px for the RGB camera and 0.137 px for the thermal camera. The intrinsic (lens distortion, focal length, field of view, image sensor offset) and relative extrinsic (position and orientation) parameters of both cameras have been pre-calibrated. Since their poses are fixed on a rotatable gimbal, the resulting image registration matrix is constant. Thus, thermal images could be computationally aligned with their corresponding RGB images after image rectification that removes lens distortion.

The entire image processing (rectification, registration, and absolute pose estimation or each drone position) was automatic and took less than 30 minutes after recording, while the subsequent image integration for visualization was real-time on a standard PC.

**Figure 3:**
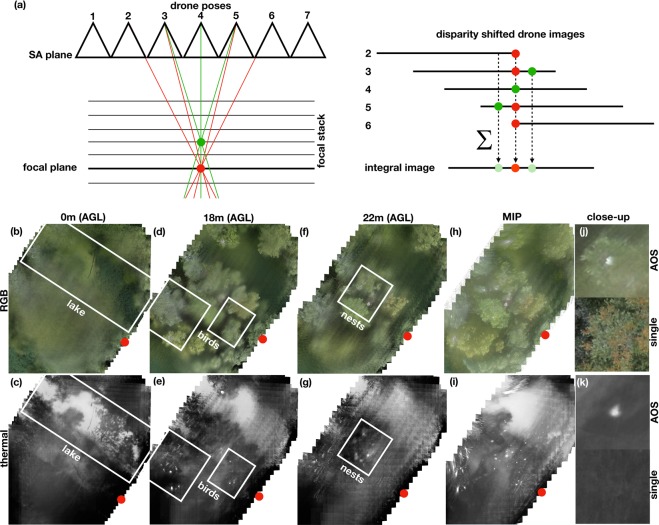
Image integration principle of AOS (**a**). Slices of the computed RGB and thermal focal stacks at different altitudes above ground level (**b–g**). Maximum intensity projection (MIP) recovers bright surface features (such as the top tree crown layer) in the RGB channel (**h**), and distinct heat sources throughout all altitudes in the thermal channel (**i**). Close-ups of occlusion case (**j,k**). The red dot indicates a common position for better orientation (same as in Fig. 1). See also Supplementary Video.

After image processing (rectification, registration, and absolute pose estimation of each drone position), we obtained a sequence of 130 aligned and geo-tagged RGB-thermal image pairs. These images were computationally integrated as explained in detail in our previous work^12^ (Fig. 3a): For a synthetic focal plane at a defined altitude, all drone images were shifted by a disparity factor which ensured that only features located on the focal plane perfectly align (red dot in Fig. 3a). Occluders at other altitudes were misaligned (green dot in Fig. 3a). The resulting integral image will therefore show targets on the focal plane clearly emphasized and in focus while occluders at different altitudes appear strongly blurred and attenuated. Thus, the wide synthetic aperture enforces an extremely shallow depth of field that quickly blurs out-of-focus occluders to reveal focused targets on the focal plane. This was repeated for a sequence of focal planes at different altitudes – ranging from the tops of the tree crowns to the forest floor. The result was two focal stacks (RGB and thermal) which optically slice the forest within an altitude range of about 26 m.

Figure 3b–g illustrates several slices of these focal stacks at different altitudes. A maximum intensity projection (MIP) mainly recovers bright surface features (such as the top tree crown layer) in the RGB channel (Fig. 3h), and distinct heat sources throughout all altitudes in the thermal channel (Fig. 3i). The example in Fig. 3j,k illustrates an occlusion case in single RGB and thermal recordings that can be recovered by AOS.

Since the temperature of birds (due to blood heat of over 40 °C) and large nests (due to composting of excrements and building material) is higher than the temperature of surrounding vegetation, they were clearly identifiable in the thermal focal stack. Note, that full or partial occlusion would not reveal them in individual recordings. After localization, the species could be identified in the RGB focal stack as it contains reflected surface color information.

**Figure 4:**
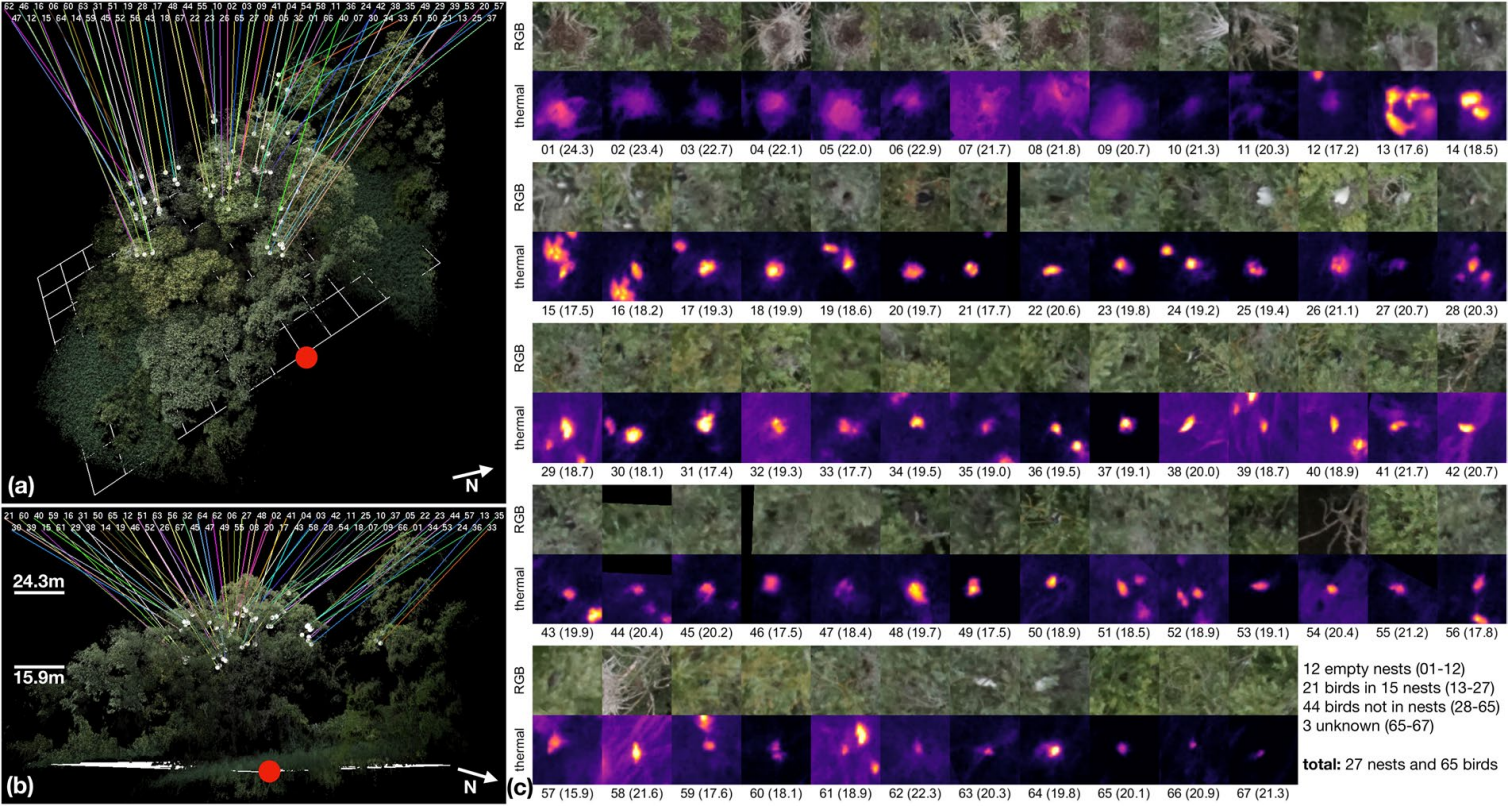
A total of 65 birds and 27 nests have been found within a 3D area of 40 × 12 × 8 m. Their 3D locations were mapped in a sparse 3D reconstruction of the forest (**a,b**). The index numbers in (**a,b**) refer to the corresponding RGB/thermal close-ups of the findspots in (**c**). The numbers in brackets is their altitudes (above ground level). The red dot indicates a common position for better orientation (same as in Figs. 1 and 3). See also Supplementary Video.

The focal stacks were interactively analyzed by an ornithologist who spotted a total of 27 nests and 65 birds (52 black-crowned night herons *(Nycticorax nycticorax)*, 5 little egrets *(Egretta garzetta)*, 8 grey herons *(Ardea cinerea)*; see also Table S1 in supplementary material for details) within the scanning area (Fig. 4c). In our recordings, 32 of them were ≥25% occluded (8 of them were ≥50% occluded). They would have been difficult to find and identify with conventional imaging. Table S1 in supplementary material provides details. The geo-tagged RGB images supported also a sparse 3D reconstruction of the forest. Although this is not directly suitable for our task since it fails in cases of considerable occlusion, the obtained coarse forest model could be used to visualize the locations of the findspots (Fig. 4a,b).

## Discussion

We demonstrated that AOS is an adequate aerial imaging technology for nesting observation. It enables recording occluded birds and nests below the tree crowns which remain invisible to normal cameras. Furthermore, it registers all recorded images to a common 3D coordinate system to support the image-based analysis of the entire forest volume. This makes bird counting much easier compared to reviewing sequential video sequences or single photographs. AOS supports 3D analysis and visualization without complete 3D reconstruction while common 3D imaging techniques, such as photogrammetry or direct laser-scanning, fail in case of dense forests with strong occlusions. It requires the same expertise and operation cost as conventional drone-assisted monitoring tasks. Thus, our technique is applicable in areas where drones are already used (or might be used in the future) by experts to monitor birds in shrubs or dense forests for ecological reasons (e.g., environmental impact assessments, monitoring endangered or invasive species, conservation or hunting regulations and laws).

Drone-assisted monitoring is less invasive and faster than traditional methods (i.e., manually traversing the natural habitat)^10^. However, it potentially introduces a new source of disturbance, especially towards birds^43^, which needs to be minimized. Small drones with low noise level motors and camouflage painting will reduce disturbance.

Furthermore, the most prominent limitation of AOS is fast motion of targets (e.g., flying or moving animals). Since the capturing process requires several minutes, they will vanish in the integral images due to motion blur. Compared to two-dimensional synthetic apertures (as applied in our field experiment), one-dimensional synthetic apertures that are sampled over a single linear scanning path reduce the capturing time down to a few seconds. This drastically minimizes motion blur while not sacrificing much quality in occlusion removal (see supplementary material for details). In future, optimal synthetic aperture scanning patterns have to be investigated that reduce motion blur while still covering large areas.

Low reflectance of sunlight and marginal heat differences between targets and surrounding environment leads to little visibility in the RGB and thermal images, and consequently in the focal stacks. Furthermore, limited battery life restricts flying time to 15 min to 30 min (depending on weight of the drone and wind conditions), which constrains the synthetic aperture size. To avoid the limitations of battery powered drones, the principles of AOS can be applied to other recording systems, as long as multiple images with overlapping field of view are recorded and a precise pose estimation is possible (e.g., planes, helicopters, boats, cars, or rail/rig systems, multiple drones scanning in parallel). Thus, larger areas, and other spectral bands might be covered.

Statistically, there exists a limit to the visibility improvement that AOS can achieve which depends on the density of the occluder volume (i.e., the forest). A maximum visibility gain is achieved at a density of 50%, with a minimum (density independent) number of images captured with a minimum (density dependent) disparity (i.e., distance of drone sampling positions)^42^.

The lateral resolution (spatial resolution on focal plane) and the axial resolution (minimal distance between two focal planes) of AOS depends on the resolution of the applied cameras, the selected focal distance (distance between synthetic aperture plane and focal plane), and the error made for pose estimation. Figure S2 in the supplementary material plots both (altitude-dependent) resolutions for our field experiment, which range from 51823 to 3224 (RGB) and 3890 to 242 (thermal) samples per square meter (lateral resolution) and 237 to 59 (RGB) and 59 to 15 (thermal) slices per meter (axial resolution).

While the preprocessing of recorded image-pairs is automatized the, classification and localization of the findspots was done manually (by ornithologists in our case), thus—as with other monitoring methods—human errors can be introduced. In the future, we also want to investigate 3D image processing and machine learning options that support a fully automatic localization and classification of birds and nests in the two focal stacks.

The application of AOS to monitor terrestrial mammals which live in dense forests, hide in shrubs, or move through high grass will also be investigated in future.

## Methods

The field experiment took place in the early morning hours of July 11th, 2019. The outside temperature was around 8 °C to 10 °C with 6/8 to 7/8 cloud coverage, 70% relative humidity, and low westerly winds (3 kn 5 kn, 230° to 240°). Flight permission over the wild life resort was granted and supervised by the authorized ornithologists.

The drone was a redundant MikroKopter OktoXL 6S12 octocopter (http://www.mikrokopter.de; 945 mm diameter; approx. 4.9 kg; purchased for €7,120). The thermal camera was a Flir Vue Pro (https://www.flir.com; purchased for €3,888) with a 9 mm fixed focal length lens and 14 bit for a 7.5 μm to 13.5 μm spectral band. The RGB camera was a Sony Alpha 6000 color camera (https://www.sony.com; purchased for €982) having a 16 mm to 50 mm lens (being set to manual operation mode and infinite focus). Both cameras were mounted axes aligned on a rotatable gimbal. The two LiPo (4500 mAh) batteries of the drone guaranteed a minimum flying time of 18 min.

The scanning path was defined in advance using MikroKopter’s flight planning software MikroKopterTool-OSD (http://wiki.mikrokopter.de/en/MikroKopterTool-OSD). The resulting waypoint protocol was then uploaded to the drone. During flight, captured images were stored on SD cards of the cameras. Both cameras were triggered synchronously by the drone as defined in the waypoint protocol. Camera synchronization was achieved with a CamCtrl control board (http://wiki.mikrokopter.de/en/CamCtrl).

After flying, all images were downloaded from the cameras and processed on personal computers. They were rectified to the same field of view of 50.82°, resampled, and cropped to resolutions of 2048 × 2048 px (RGB) and 512 × 512 px (thermal), which took around 70 s (including reading and writing to files). We applied OpenCV’s (https://opencv.org) camera model for camera calibration, image rectification, and image registration.

For drone pose estimation with the RGB images and sparse 3D reconstruction of the forest, the general-purpose structure-from-motion and multi-view stereo pipeline, COLMAP^36^ (https://colmap.github.io), was used and took approx. 25 min (including reading and writing to files).

Focal stack rendering, visualization of the sparse 3D forest model, interactive navigation and selection tools to mark individual findspots in the focal stacks, and the labeling in the 3D forest model was implemented on the GPU, based on Nvidia’s CUDA toolkit (https://developer.nvidia.com/cuda-downloads) and runs in realtime (i.e., rendering of one frame takes 62.5 ms).

The 130 recorded images pairs (thermal and RGB), the pose estimation data (in standard data formats), and the computed focal stacks are available with open access^44^.

## Supplementary information

Supplementary Information.

Supplementary Video
