# Supplementary material for: Airborne Optical Sectioning for Nesting Observation
Source: Sci Rep. 2020 Apr 29;10:7254. doi: 10.1038/s41598-020-63317-9 (PMC7190638; doi:10.1038/s41598-020-63317-9)
Supplement: Supplementary file 1 — Supplementary Information. [file 41598_2020_63317_MOESM1_ESM.pdf]

Airborne Optical Sectioning for Nesting Observation

(Supplementary Material)

David C. Schedl, Indrajit Kurmi, and Oliver Bimber

Site Details and Ornithologist's Analysis

The local ornithologists keep a running record[1,2] of the number of occupied nests by observation with conventional camera drones. The nesting area is surrounded by wetlands. In the year 2019, for example, two conventional camera drone flights have been carried out in early spring (before leaves are growing) and in autumn (after the trees lose their leaves), which helped to accurately count the nests. The nest count was used to estimate the population of adults. According to the ornithologist, birds did not react to the light-weight drone they where using. In total, approx. 30 grey heron nests and 55 to 60 smaller nests (i.e., black-crowned night heron and little egret nests) have been observed, in 2019. Note, that the numbers differ from Table S1, as we only recorded a fraction of the total nesting site.

Aerial imaging during the actual breading season (March - July) in which young and adult animals are more stationary in their nests and could therefore be directly counted, however, was not possible before because of dense leaf occlusion.

On July 24, 2019 the AOS data (recorded on July 11, 2019) was analyzed by an ornithologist who interactively browsed through the focal stacks. The classification of the 67 findspots took around 1.5 h. Table S1 summarizes all findings for the recorded area. The visibility in Table S1 indicates how likely it is to detect the birds and nests in conventional single thermal images if a 2D pattern or a line pattern (1D) is used for recording. Note, however, that the localization (i.e., the altitude) of findspots is not possible without AOS. The altitude of a findspot helped the ornithologist during classification (e.g., some species reside on lower branches). The feedback of the expert (the ornithologists continuously observing the nature reserve in Reichersberg) was that AOS is well suited for nesting observation – in particular because it enables to count nesting birds directly during breading season.

All three observed species (i.e., black-crowned night herons *(Nycticorax nycticorax)*, little egrets *(Egretta garzetta)*, and grey herons *(Ardea cinerea)*) are categorized as endangered (EN) on the Upper Austrian Red List of Threatened Species[2]. Thus, a constant monitoring of the population is essential for preservation of the species, and allows for proper counter measurements if population sizes change (e.g., loss of habitat, predators, or climate change). Furthermore, while the black-crowned night herons and little egrets are protected by the Nature Conservation Law, grey herons are a hunted species, in Austria. A precise population count can also be the foundation for an ecological hunting regulation.

ID	bird (count and species)	altitude (AGL)	description	views (visibility)	1D scan (visibility)
1	0	24.3 m	empty grey heron nest	13 (92 %)	4 (100 %)
2	0	23.4 m	empty grey heron nest	22 (95 %)	6 (100 %)
3	0	22.7 m	empty grey heron nest	23 (65 %)	7 (71 %)
4	0	22.1 m	empty grey heron nest	30 (100 %)	7 (100 %)
5	0	22.0 m	empty grey heron nest	20 (50 %)	5 (60 %)
6	0	22.9 m	empty grey heron nest	5 (60 %)	0
7	0	21.7 m	empty grey heron nest	29 (83 %)	7 (86 %)
8	0	21.8 m	empty grey heron nest	26 (38 %)	7 (43 %)
9	0	20.7 m	empty grey heron nest	20 (100 %)	5 (100 %)
10	0	21.3 m	empty grey heron nest (new, due to low composting)	13 (77 %)	2 (100 %)
11	0	20.3 m	empty grey heron nest (old; probably unused)	40 (78 %)	9 (78 %)
12	0	17.2 m	empty grey heron nest	14 (79 %)	0
13	3 grey heron	17.6 m	(probably young animals) in nest	12 (100 %)	3 (100 %)
14	2 grey heron	18.5 m	in nest	16 (75 %)	1 (100 %)
15	2 grey heron	17.5 m	(adult) spreading wings and (young) in a nest	29 (93 %)	5 (100 %)
16	1 grey heron	18.2 m	in nest (new nest, due to low composting)	27 (89 %)	5 (100 %)
17	2 black-crowned night heron	19.3 m	(young) in a nest (probably grey heron nest, due to nest size)	14 (79 %)	1 (100 %)
18	2 black-crowned night heron	19.9 m	in nest	6 (83 %)	0
19	1 black-crowned night heron	18.6 m	in nest	19 (74 %)	3 (100 %)
20	1 black-crowned night heron	19.7 m	in nest	35 (89 %)	6 (100 %)
21	1 black-crowned night heron	17.7 m	(young) in nest (probably grey heron nest, due to nest size)	18 (72 %)	0

22	1 black-crowned night heron	20.6 m	(adult) or a young grey heron, close to nest	4 (100 %)	0
23	1 black-crowned night heron	19.8 m	in nest (probably grey heron nest, due to nest size)	4 (75 %)	0
24	1 black-crowned night heron	19.2 m	(young) in nest (probably grey heron nest, due to nest size)	27 (78 %)	6 (83 %)
25	1 little egret	19.4 m	(probably young) in nest	29 (76 %)	2 (100 %)
26	1 little egret	21.1 m	in nest	29 (79 %)	7 (71 %)
27	1 little egret	20.7 m	very likely in nest	30 (67 %)	7 (43 %)
28	4 black-crowned night heron	20.3 m	3 young and 1 adult	6 (100 %)	0
29	2 black-crowned night heron	18.7 m	(young)	27 (85 %)	3 (33 %)
30	2 black-crowned night heron	18.1 m	(probably adult)	29 (93 %)	4 (50 %)
31	2 black-crowned night heron	17.4 m	probably (hard to identify)	34 (88 %)	7 (100 %)
32	2 black-crowned night heron	19.3 m	probably (hard to identify)	39 (67 %)	8 (63 %)
33	1 black-crowned night heron	17.7 m	(probably young)	6 (67 %)	1 (100 %)
34	1 black-crowned night heron	19.5 m	(probably young)	30 (90 %)	7 (86 %)
35	1 black-crowned night heron	19.0 m	(probably young)	4 (75 %)	0
36	1 black-crowned night heron	19.5 m	(probably young)	23 (91 %)	5 (100 %)
37	1 black-crowned night heron	19.1 m	(probably young)	44 (86 %)	9 (78 %)
38	1 black-crowned night heron	20.0 m		20 (100 %)	0
39	1 black-crowned night heron	18.7 m	probably (hard to identify)	13 (54 %)	0
40	1 black-crowned night heron	18.9 m	(adult)	29 (79 %)	4 (100 %)
41	1 black-crowned night heron	21.7 m	(young)	19 (47 %)	3 (33 %)
42	1 black-crowned night heron	20.7 m	(young)	34 (100 %)	8 (100 %)
43	1 black-crowned night heron	19.9 m		11 (82 %)	1 (100 %)
44	1 black-crowned night heron	20.4 m		3 (100 %)	0
45	1 black-crowned night heron	20.2 m		13 (77 %)	1 (100 %)
46	1 black-crowned night heron	17.5 m		12 (92 %)	0
47	1 black-crowned night heron	18.4 m	probably (hard to identify)	9 (67 %)	0
48	1 black-crowned night heron	19.7 m	(adult) or a young grey heron	22 (59 %)	2 (50 %)
49	1 black-crowned night heron	17.5 m	(young)	44 (80 %)	8 (100 %)
50	1 black-crowned night heron	18.9 m	(adult)	24 (92 %)	0
51	1 black-crowned night heron	18.5 m		18 (83 %)	2 (100 %)
52	1 black-crowned night heron	18.9 m		17 (82 %)	2 (100 %)
53	1 black-crowned night heron	19.1 m	(young)	26 (23 %)	3 (0 %)
54	1 black-crowned night heron	20.4 m	(young)	36 (78 %)	8 (88 %)
55	1 black-crowned night heron	21.2 m	(young)	22 (55 %)	5 (80 %)
56	1 black-crowned night heron	17.8 m		18 (56 %)	2 (100 %)
57	1 black-crowned night heron	15.9 m	(young)	31 (19 %)	5 (20 %)
58	1 black-crowned night heron	21.6 m	(young)	31 (29 %)	8 (0 %)
59	1 black-crowned night heron	17.6 m	probably (hard to identify)	34 (82 %)	7 (86 %)
60	1 black-crowned night heron	18.1 m	(young)	32 (53 %)	6 (67 %)
61	1 black-crowned night heron	18.9 m		22 (50 %)	1 (0 %)
62	1 black-crowned night heron	22.3 m	probably (hard to identify)	4 (25 %)	0
63	1 little egret	20.3 m		11 (64 %)	0
64	1 little egret	19.8 m		9 (100 %)	0
65	0 unknown	20.1 m	not identifiable	29 (69 %)	5 (100 %)
66	0 unknown	20.9 m	not identifiable	28 (43 %)	7 (43 %)
67	0 unknown	21.3 m	not identifiable	24 (46 %)	5 (80 %)

Table S1. Results of ornithologist's analysis of findspots: ID as in Fig. 4 of the main paper, bird count and species, altitude (above ground level), description, for 2D and 1D synthetic apertures: maximum number of recorded images from which findspot was within the camera's field-of-view (number of recorded images, in %, in which findspot was visible / not occluded).

Lateral and Axial Resolutions

The lateral sampling resolution f_l (number of samples per unit area) at the focal plane can be determined by back-projecting the focal plane onto the imaging plane of the drone's camera (i.e., the synthetic aperture plane)[3]. Assuming a pinhole camera

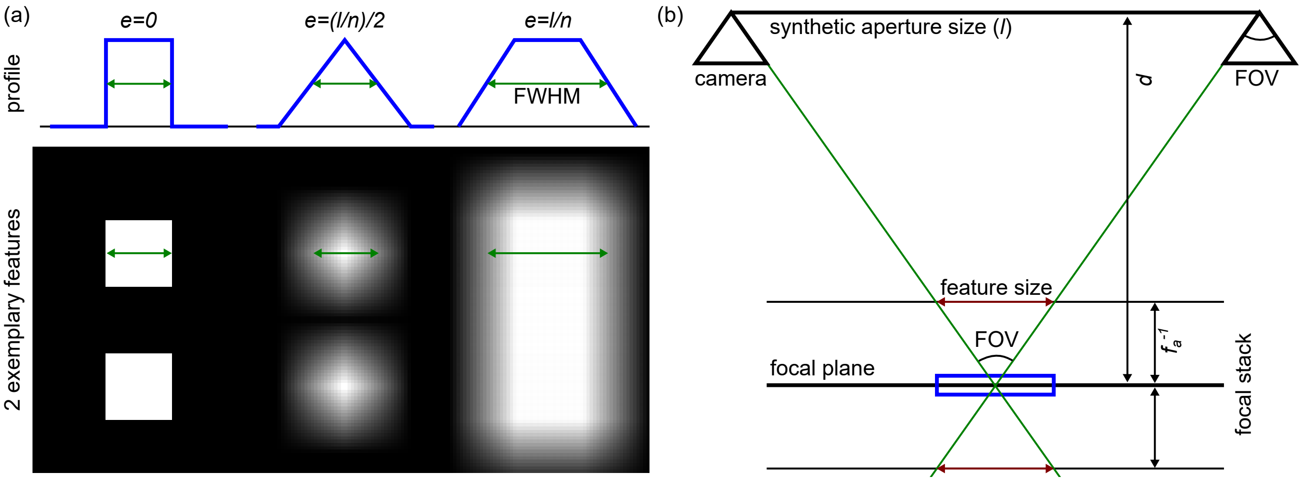

Figure S1. The lateral resolution (a) under consideration of a pose-estimation error (e). As long as the pose estimation error is smaller than the half feature size ($2e \leq l/n$) the Full Width at Half Maximum (FWHM) is low (columns 1 and 2). If the pose-estimation error exceeds the half-feature-size limit the FWHM gets large and features are blurred (column 3). As the FWHM becomes larger, neighboring features cannot be distinguished. The axial resolution (b) is influenced by the field of view (FOV), the distance between the focal plane and the synthetic aperture plane (d), and the size of features in focus. Note, that the aperture size (l) is determined by the FOV and d, and is the same as the resolvable area on the focal plane.

model, parallel focal and image planes, and a square sensor this leads to:

$$f_l(d) = \frac{n^2}{l(d)^2}, \tag{1}$$

where n is the camera's spatial resolution (number of pixels on one axis of a square image sensor), d the distance between focal plane and the synthetic aperture plane, $l(d) = 2d\tan(\mathrm{FOV}/2)$ the side length of the area in view on the focal plane, and FOV the camera's field of view[3]. In the presence of a pose-estimation error, features are misaligned during image integration. This results in an increased and blurred feature radius, as shown in Fig. S1a. Mathematically, this can be expressed as a box function (the feature) which is convolved with another box function (the pose uncertainty). This leads to a triangle or truncated triangle shaped profile of the feature. For measuring the sampling resolution, we apply the Full Width at Half Maximum (FWHM) metric. This metric is commonly used for measuring optical resolution limits (e.g., in microscopy). Furthermore, the FWHM is well defined for a triangular profile: the maximum width of the two box functions (i.e., the feature size (l/n) and the pose uncertainty). Thus, equation (1) extents to

$$f_l'(d) = \frac{1}{\max\left(\sqrt[-2]{f_l(d)}, 2e(d)\right)^2}, \tag{2}$$

where $e(d)$ is the average pose-estimation error on the focal plane at distance d[3].

The axial resolution defines the minimal distance between two focal planes which causes a distinguishable difference in the resulting images after integration. Similar as for a pose-estimation error, the image integration can be considered as a convolution of two box functions (i.e., the feature and the synthetic aperture projected onto the focal plane as shown in Fig. S1b).

Thus, by applying the FWHM metric again, the axial resolution $f_a(d)$ (the number of focal slices per unit distance) equals

$$f_a(d) = \frac{l(d)}{\max\left(\frac{l(d)}{n}, 2e(d)\right) d}, \tag{3}$$

where the synthetic aperture size equals the size of the synthetic aperture plane projected onto the focal plane.

For our field experiment, they range from 51 823 to 3224 (RGB) and 3890 to 242 (thermal) samples per square meter (lateral resolution) and 237 to 59 (RGB) and 59 to 15 (thermal) slices per meter (axial resolution). Note that the resolution of the RGB camera is limited by the pose-estimation error of 0.548 px, as the error exceeds half the size of a pixel. For the thermal

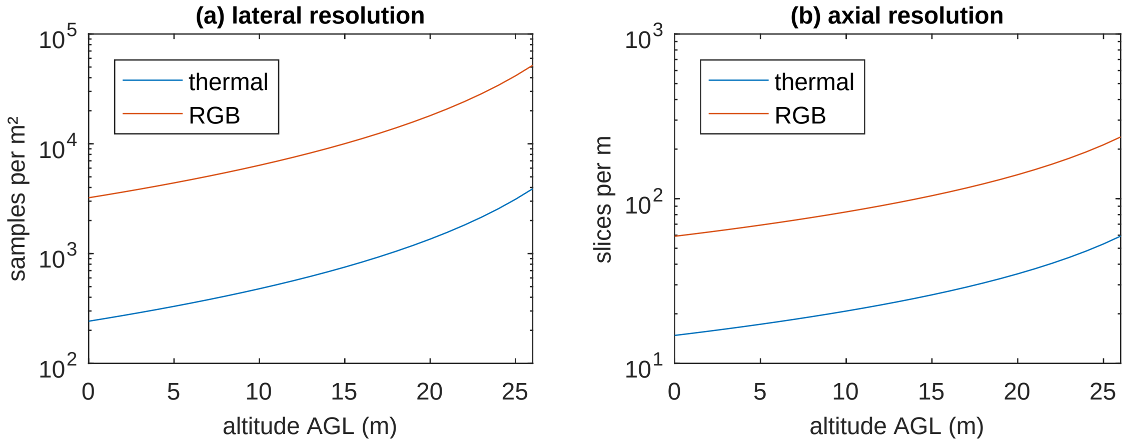

Figure S2. The lateral resolution (a) and the axial resolution (b) of the thermal and RGB camera computed with equations (2) and (3) for the focal stack as shown in Fig. 3. Note, that the vertical axes of these plots have logarithmic scaling.

camera the pose-estimation error does not degrade the resolution as the pose estimation is below half a pixel size (0.137 px). Figure S2 plots the spatial and axial resolutions of RGB and thermal cameras for our field experiment using equations (2) and (3). Note, that both resolutions vary with altitude (distance (d) between synthetic aperture plane and focal plane). Therefore, we plot them as a function of altitude above ground level (AGL). Note, that both resolutions decrease with an increasing distance d (i.e., lower altitude AGL).

One-dimensional vs. Two-Dimensional Synthetic Aperture

Figure S3 and Table S1 compare AOS results of a one-dimensional (40 m) synthetic aperture to the results achieved with the full two-dimensional (40 m $\times$ 12 m) synthetic aperture actually being measured in our field experiment. For this, we chose only a subset of sampling positions (center scanline from waypoint 7 to waypoint 8 in Fig. 1c of the main paper).

The 2D aperture was recorded in a 2-m-spaced grid-like pattern, line by line, within 7 min and resulted in a total of 130 image pairs. Thus, findspots might be in the field of view from multiple scan lines, which increases their recording interval (number of views in which a findspot is within the field of view). In our field experiment, 3 min 15 s were required on average to fully record all views of a single findspot (on average 21.5 views over 4.3 out of 7 scan lines). A recording over 3 min, however, will cause significant motion blur in case birds move during this time.

The 1D aperture, in contrast, samples only along a single scan line of approx. 40 m. This shortens the total flight time to 55 s for recording 20 image pairs. This reduces the recording interval to 4.8 views, and the recording time to 9 s per findspot on average. While the latter significantly improves motion blur of moving targets, the former does not lead to significantly worse results: Since the total field of view of the 1D aperture is smaller than the one of the 2D aperture, only 50 out of the 67 findspots were actually captured (the remaining were entirely out of the field of view at all sampling positions). But only 3 findspots that were detectable with the 2D aperture could not be identified with the 1D aperture due to occlusion (ID: 53, 58, 61 in Table S1). Furthermore, 9 findspots are visible in only one view (ID: 14, 17, 29, 33, 41, 43, 45, 48, 57 in Table S1), which makes it harder to reliably localize them in 3D, as at least two views are required for triangulation. For most cases, however, nearby features can be used to estimate the altitude.

For a quantitative comparison, we calculated the mean squared error (MSE) between thermal and RGB focal stacks computed for the 2D and for the 1D synthetic apertures over the range of 0 m to 26 m altitude above ground level. For normalized RGB and thermal values, it was 0.0021 and 0.0020, respectively.

With respect to Fig. S3, the difference between 1D and 2D apertures is higher when fewer views are available for image integration. Since less views overlap, artifacts and differences are most noticeable at high altitudes (i.e., close to the synthetic aperture plane). Focal stack slices close to the ground exhibit less noticeable differences, due to the high number of available views. Furthermore, since the bokeh of defocused features corresponds to the shape of the synthetic aperture (scaled proportionally to the amount of defocus), it changes from a 2D box-shaped to 1D line-shaped bokeh.

For the 1D aperture, parallax is recorded only in one direction, thus equation (3) can only be applied to one axis. The lateral and axial resolutions of AOS (equations (2) and (3)), however, remain the same.

All of this suggests, that single linear scanning paths with an adequate high number of samples are beneficial for reducing motion blur in case of quickly moving targets. It has been statistically proven, that the visibility gain AOS can achieve depends on the density of the occluder volume (i.e., the forest) and the number of recorded samples, but is independent of the aperture

shape[4]. Only a (density-depended) minimum spacing of sampling views has to be guaranteed. Increasing the number of recording samples does not increase the scanning time significantly, as image capturing is literally done on the fly. The scanning path only has to be extended to 2D for capturing a larger area.

Unprocessed Image Material

Figures S4 and S5 illustrate the unprocessed 8 bit RGB and color coded 14 bit thermal images as captured by the drone during the field experiment. The original 130 recorded images pairs are available online[5], together with the pose estimation data that can be used in various software tools (e.g., Colmap or Meshlab), and the computed focal stacks of Fig. 3.

References

1. Brader, M. & Aubrecht, G. *Atlas der Brutvögel Oberösterreichs. Denisia 7.* 194 (Ornithologische Arbeitsgemeinschaft am Oberösterreichischen Landesmuseum, 2001).

2. *Atlas der Brutvögel Oberösterreichs 2013-2018* (Ornithologische Arbeitsgemeinschaft am Oberösterreichischen Landesmuseum, 2020). In press.

3. Kurmi, I., Schedl, D. C. & Bimber, O. Airborne optical sectioning. *J. Imaging* **4**, DOI: 10.3390/jimaging4080102 (2018).

4. Kurmi, I., Schedl, D. C. & Bimber, O. A statistical view on synthetic aperture imaging for occlusion removal. *IEEE Sensors J.* 1–1, DOI: 10.1109/JSEN.2019.2922731 (2019).

5. Schedl, D., Kurmi, I. & Bimber, O. Data: Airborne optical sectioning for nesting observation. *figshare* https://figshare.com/articles/Data_Airborne_Optical_Sectioning_for_Nesting_Observation/11836296 (2020).

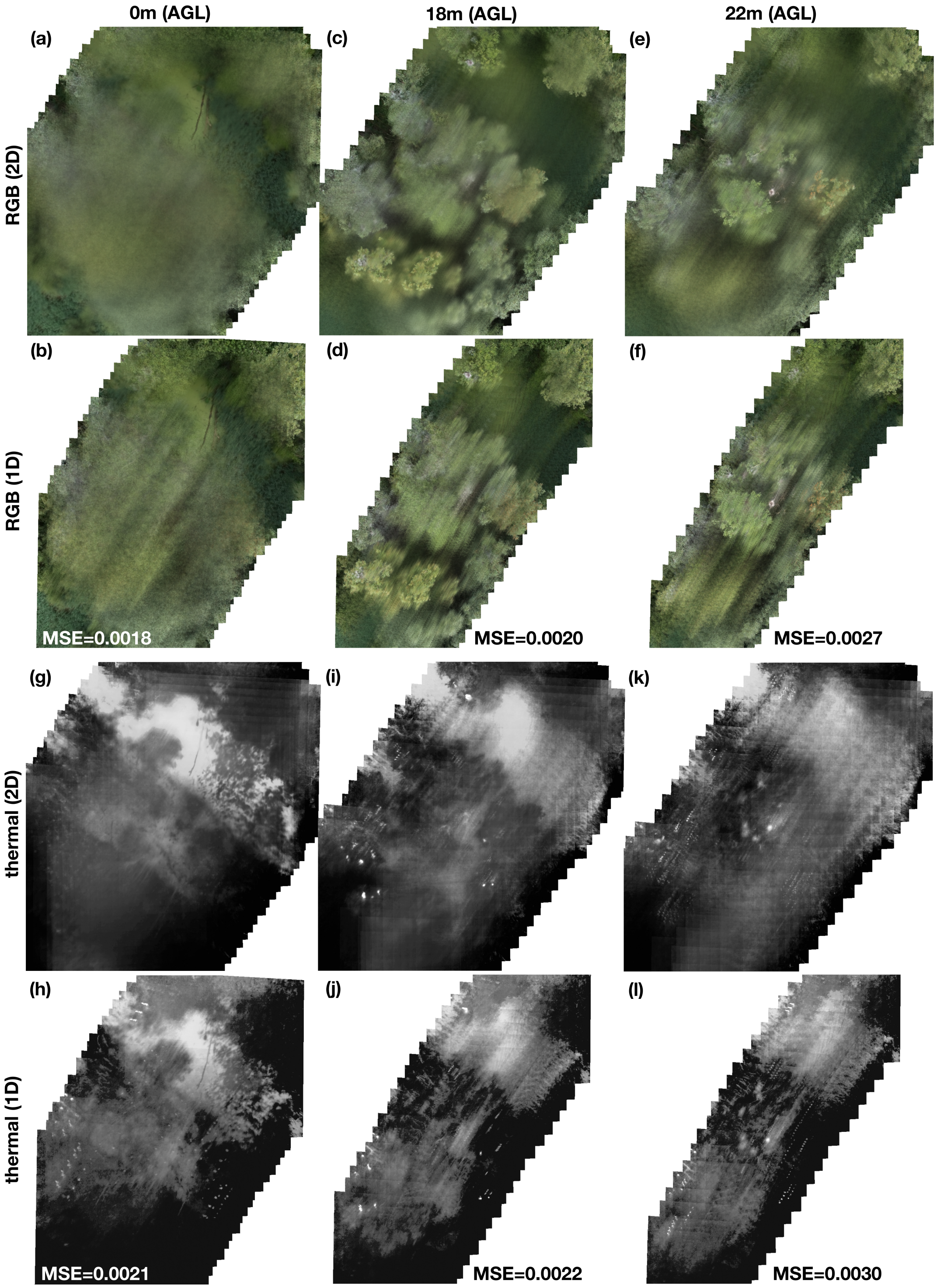

Figure S3. Comparison between two-dimensional (40 m × 12 m, 130 image pairs) and one-dimensional (40 m, 20 image pairs) synthetic aperture. RGB (a–f) and thermal (g–l) focal stack slices at same altitudes (AGL), with 2D aperture (a,c,e,g,i,k) and 1D aperture (b,d,f,h,j,l) The mean squared error (MSE) indicates the quantitative difference between corresponding 1D and 2D focal stack slices.

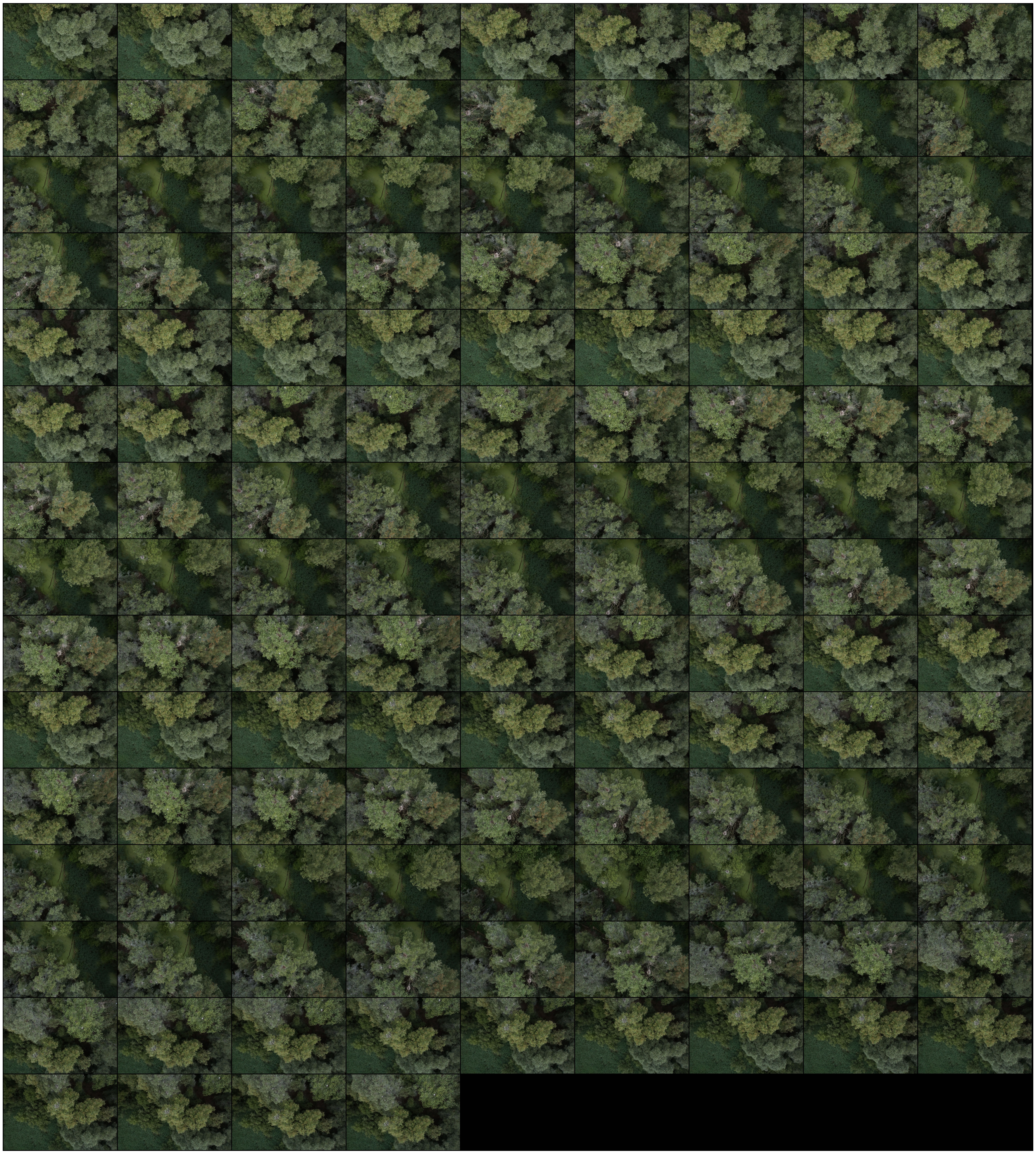

Figure S4. Unprocessed 8 bit RGB images captured during the field experiment (from top left to bottom right: position 1 to position 14 in Fig. 1c).

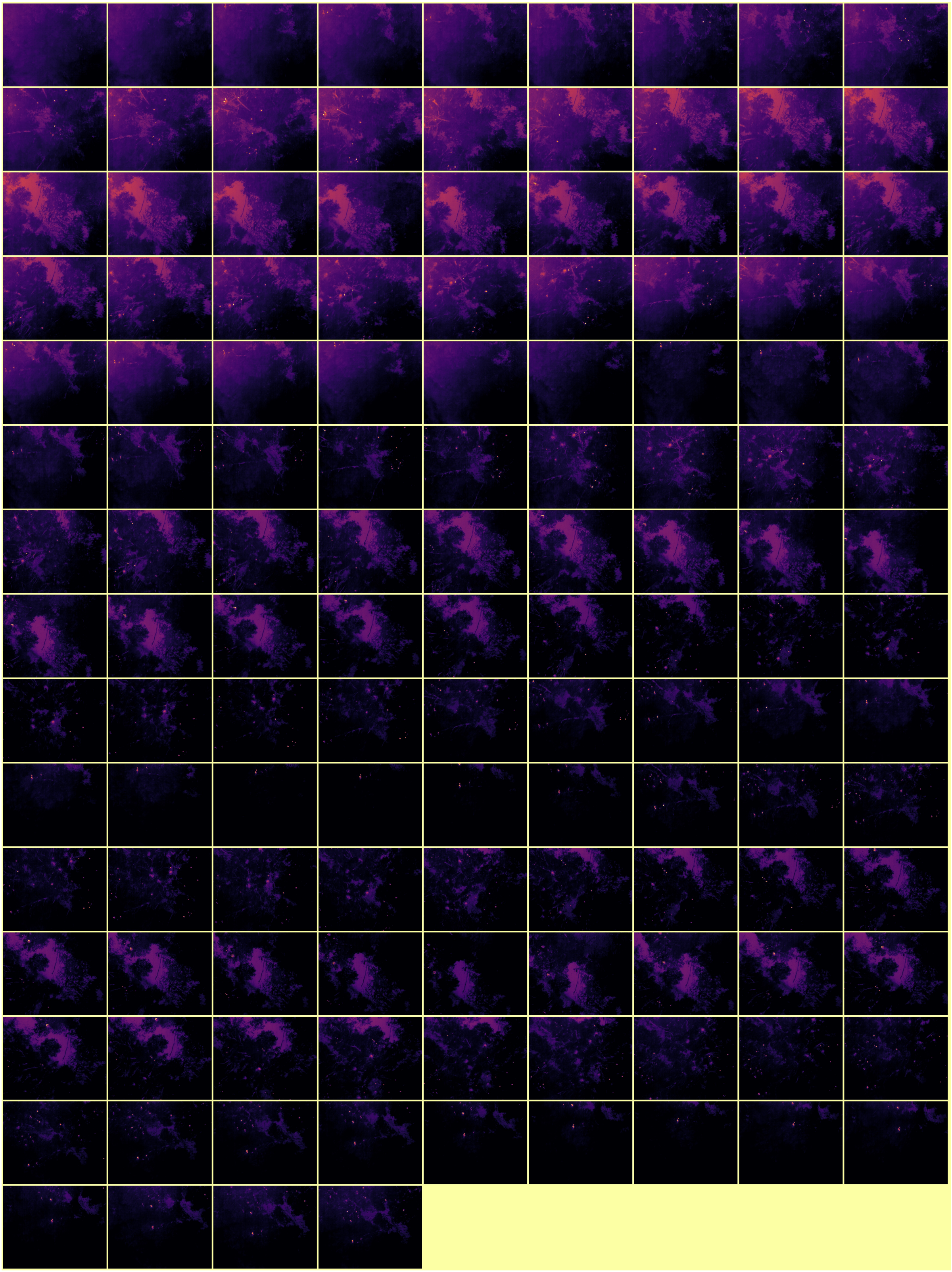

Figure S5. Unprocessed color coded 14 bit thermal images captured during the field experiment (from top left to bottom right: position 1 to position 14 in Fig. 1c).
